# Recruitment progression rules for internal pilot studies monitoring recruitment

**DOI:** 10.1186/1745-6215-16-S2-O89

**Published:** 2015-11-16

**Authors:** Lisa Hampson, Paula Williamson, Martin Wilby, Thomas Jaki

**Affiliations:** 1 Lancaster University, Lancaster, UK; 2 MRC North-West Hub for Trials Methodology Research, University of Liverpool, Liverpool, UK; 3 Walton Centre NHS Foundation Trust, Liverpool, UK

## 

Just over half of publicly funded trials recruit their target sample size within the planned study duration. When recruitment targets are missed, the funder of a trial is faced with the decision of either committing further resources to the study or risk that a worthwhile treatment effect may be missed by an underpowered final analysis. To avoid this challenging situation, when there is insufficient prior evidence to support predicted recruitment rates funders now require feasibility assessments to be performed in the early stages of trials. Progression criteria are usually specified and agreed with the funder ahead of time. To date, however, the progression rules used are typically ad hoc. In addition, rules routinely permit adaptations to recruitment strategies but do not stipulate criteria for evaluating their effectiveness. In this presentation we consider internal pilot studies which permit a trial to be stopped early if recruitment is disappointing or to continue to full recruitment if enrolment during the feasibility phase is adequate. We propose novel two-stage designs which stipulate that if neither of these situations arises, adaptations to recruitment should be made and subsequently evaluated to establish whether they have been successful. We derive optimal progression rules for internal pilot studies that can be specified in advance which minimise the expected trial overrun and maintain a high probability of completing the study when the recruitment rate is adequate. The advantages of this procedure are illustrated using a real trial example.

